# Developmental competence of immature oocytes aspirated from antral follicles in patients with gynecological diseases

**Published:** 2015-08

**Authors:** Fereshteh Safian, Mohammad Ali Khalili, Mojgan Karimi-Zarchi, Mehdi Mohsenzadeh, Sareh Ashourzadeh, Marjan Omidi

**Affiliations:** 1 *Department of Biology and Anatomical Sciences, Shahid Sadoughi University of Medical Sciences, Yazd, Iran.*; 2 *Research and Clinical Center for Infertility, Shahid Sadoughi University of Medical Sciences, Yazd, Iran.*; 3 *Department of Obstetrics and Gynecology, Faculty of Medicine, Shahid Sadoughi University of Medical Sciences, Yazd, Iran.*; 4 *Afzalipour Clinical Center for Infertility, Afzalipour Hospital, Kerman University of Medical Sciences, Kerman, Iran.*

**Keywords:** *Ovarian follicle*, *IVM*, *Human oocytes*, *Fertility preservation*

## Abstract

**Background:**

In vitro maturation (IVM) of immature oocytes collected from ovary has been proposed for fertility preservation. In addition, quality of oocytes post IVM is one of the factors determining its developmental competence. By using the non-invasive Polscope system, both meiotic spindle (MS) and zona pellucida (ZP) can be assessed in living oocytes.

**Objective:**

The aim was to investigate the developmental potential of immature oocytes retrieved from ovarian tissue after IVM, as a method for fertility preservation, in patients with gynecological diseases.

**Materials and Methods:**

The ovarian cortex from 26 patients with malignant and benign diseases (21-45 years old), were obtained directly from collaborating hospitals, and transported to the IVF center on ice. In total 61 immature oocytes were aspirated, of which 18 (29.5%) were degenerated and discarded. The remaining 43 (70.5%) healthy oocytes were cultured in IVM culture media for 48 hr. The rate of maturity was assessed, and the ZP birefringence and MS were imaged with Polscope technology.

**Results:**

Overall 43 immature oocytes underwent IVM technology, of which 30.2% reached viable metaphase II (MII) oocytes. The ovarian tissues of 9 (34.6%) women were lacking oocytes at any stage. During polarized light microscopy examination, MS could be visualized only in one of the MII oocytes, but high ZP birefringence’s were observed in the majority of the oocytes post IVM (61.5%).

**Conclusion:**

Oocytes maturation post IVM from unstimulated ovaries showed a good developmental competence in gynecologic patients. Further studies should be performed to advance the oocyte maturation program, such as co-culture system, for fertility preservation.

## Introduction

Over the last decades, early detection and advances in the treatment of women diagnosed with cancer in their reproductive age have markedly increased (1). However, the effects of cancer and its treatment on fertility has emerged as a major quality of life issue for cancer survivors (2). For cancer patients, several strategies have been proposed as options for preserving fertility (3). Nowadays, in developed countries, women for financial or social reasons delay child bearing until later in life. So, the numbers of women that diagnosed with cancer in reproductive age who are interested in preserving fertility are increasing (4, 5). There are several methods for preserving the reproductive potential prior to gonadotoxic treatment, such as cryopreservation of their immature or mature oocytes, embryo and ovarian tissue (6, 7).

Recently, some studies have suggested an in vitro maturation (IVM) of immature oocytes collected from excised ovary followed by oocyte vitrification in patients undergoing ovarian cryobanking. This can be considered as good alternative methods for fertility preservation particularly for pre-pubertal girls or single women (1, 8). It provides shorter time to oocyte collection not only required hormone pre-treatment or delayed for initiation of cancer treatment (9, 10). Originally, IVM program provides a milder treatment for infertility (11). For current practice, oophorectomy can be performed for the purpose of fertility preservation prior to toxic treatment and aspirated oocytes can be cultured in vitro (12, 13).

The oocyte quality or morphology may be one of the factors determining its developmental competence and outcome of infertility treatment protocols (14). Moreover, anatomical structures of the IVM oocytes will be assessed by using the Polscope system, which does not require invasive techniques, such as fixation or staining (15). Polarized light imaging can analyze birefringent characteristics, such as the meiotic spindle (MS) and zona pellucida (ZP) in living oocytes (16). Therefore, this study aimed to evaluate the immature oocytes from ovaries removed by laparotomy or total abdominal hysterectomy (TAH) in gynecologic patients without any stimulation, then the efficiency of maturated oocytes was assessed in IVM program.

## Materials and methods

**Patients**

In this experimental study, the excised ovarian tissues and large biopsies of the ovarian cortex from 26 patients (mean age 34.03±7.3 years) with malignant and benign gynecological diseases were removed. Out of these patients, 7 were diagnosed with squamous metaplasia, 2 cervical neoplasia, 2 papillary serous carcinoma, 7 Leiomyoma, 2 mature cystic teratoma, and 6 with other types of ovarian complications including mucinous, serous, and endometriotic cystic. The investigation took place from Dec 2013 to Dec 2014 at Yazd Institute for Reproductive Sciences, Yazd, Iran. The inclusion criteria were: age (21-45 years), partial ovarian tumor, and no prior chemotherapy. The exclusion criteria included: complete ovarian tumor or being under chemotherapy. In addition, in all recruited patients, the day of menstrual cycle and history of infertility were ignored. Ovarian samples were obtained from collaborating hospitals and transported to the research laboratory in Hams’ F10+HAS medium at 4^°^C within 30 min. The study was approved by Ethics Committee of Research and Clinical Center for Infertility, Yazd, Iran.

**Oocyte retrieval**

All visible antral follicles on the surface of ovaries were aspirated using scalp vein G21 gauge needles under pressure of 40-50 mm Hg. Then, using stereo microscope, the aspirated follicular fluid (FF) was examined for cumulus oocyte complexes (COCs). The immature oocytes were examined for meiotic stage and denuded oocytes were washed three times in pre-warmed IVM washing medium. Then, aspirated oocytes were placed in humidified incubator at 37^°^C with 5% CO2 and 95% air for 48 hr. The ovarian tissues of 9 women were lacking oocytes at any stage.

**In vitro maturation**

Immature oocytes were subsequently transferred to pre-equilibrated IVM medium consisting of: Ham’s F10 (Biochrom Co, Germany) supplemented with 0.75 IU LH, 0.75 IU FSH (Ferring Co, Germany) and 40% FF, as described previously (17). Briefly, FF was centrifuged at 3,500 rpm for 10 min to remove granulosa and blood cells, then inactivated in water bath at 56^°^C for 30 min. Pure HFF was filtered with a 0.22 μm filter, and aliquoted and stored at -20^°^C (18). Oocyte maturation was confirmed under an inverted microscope (Nikon Co, Japan) after 24 hr and 48 hr by the presence of first polar body.

**Morphologic observations and imaging of ZP and MS**

The morphology of in-vitro matured oocytes was evaluated under inverted microscope (TE300; Nikon, Japan) with a heated stage. The oocytes morphologic characteristics were determined by variables of irregular shape, presence of vacuoles, appearance of smooth endoplasmic reticulum clusters (SERc), refractile bodies (RF), wide perivitelline space (PVS), and fragmented polar body (fPB) (19-21). For polarized light microscopy, each oocyte was placed on a glass bottom dish (Wilkos, Netherlands) in a droplet of buffered medium (G-Mops-V1; Vitrolife) overlaid with mineral oil (Irvine Scientific, USA) and kept on a 37^°^C stage under the polarization microscope (OCTAX PolarAIDE; Octax). Immediately, the oocytes were screened for visualization of the MS and concentration and homogeny of the inner layer of ZP.

**Statistical analysis**

The data were presented as mean±SD and percentage for qualitative data. Linear correlation test was applied for analyzing data between age and number of collected oocytes (Pearson, p<0.05). Statistical evaluation was done using the SPSS software (Statistical Package for the Social Sciences version 20.0, SPSS Inc., Chicago, IL, USA).

## Results

**IVM outcomes of immature oocytes**

**Figure 1:**
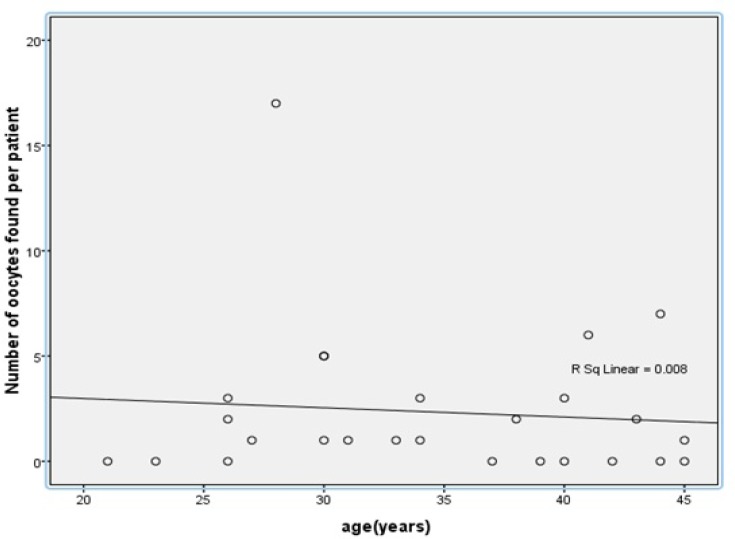
Relationship between the age of the patients and the number of oocytes retrieved from ovarian tissue. Linear regression line. y=3.862_0.09x, R2=0.008 (p>0.05).

In total, 61 immature oocytes from 26 patients were retrieved. Only 13 (30.2%) oocytes reached maturity and extruded first polar body. Furthermore, immature oocytes that reached the MII stage were enclosed by cumulus cells. In 9 (34.6 %) women, no oocytes were found. Also, 18 (29.5%) of the aspirated oocytes were degenerated at the time of aspiration. The remaining 43 (70.5%) healthy aspirated oocytes were classified according to the oocyte nuclear stage as germinal vesicle (GV), and germinal vesicle breakdown (MI). In all, 34 (79%) immature oocytes were considered as GV, and 9 (21%) were MI. Also, retrieval of immature oocyte that underwent IVM was performed in seven groups of patients. The maturation rates, the mean age of patients and the median number of aspirated oocytes in any types of diseases are presented in table 1. The median number of aspirated oocytes and maturation rate in patients with cervical neoplasia was higher than other groups. The findings also showed there was no correlation between patients age and the number of retrieved oocytes (R=0.09, n=26; p>0.05) (Figure 1). In addition, almost half of the patients were over age of 38 years.

**Morphologic observations**

The data reveal that after IVM, the most frequent abnormalities in MII oocytes was wide PVS (36.3%), and dark cytoplasm (27.2%). Furthermore, the fPBs were observed in 38.4 percent of the matured oocytes. In addition, oocytes retrieved from one patient (30 years old) were found to have large sized irregular shape. 

**MS and ZP birefringence examination**

**Figure 2:**
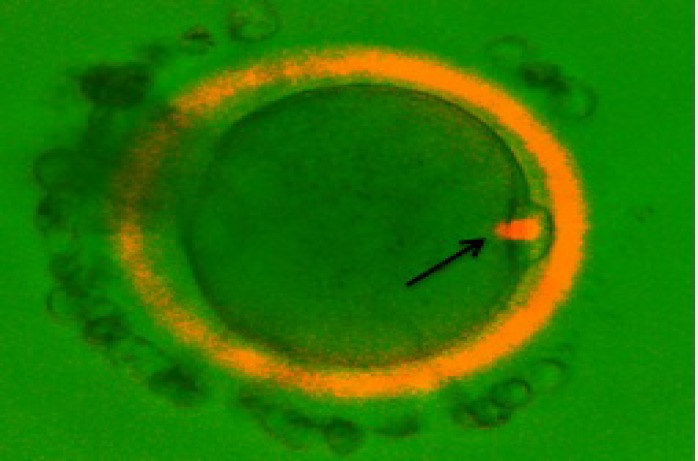
PolScope (OCTAX PolarAID; Octax, Herbon, Germany) image of an IVM human oocyte. A bright meiotic spindle (arrow) is clearly visible. ( ×40 magnification).

**Figure 3:**
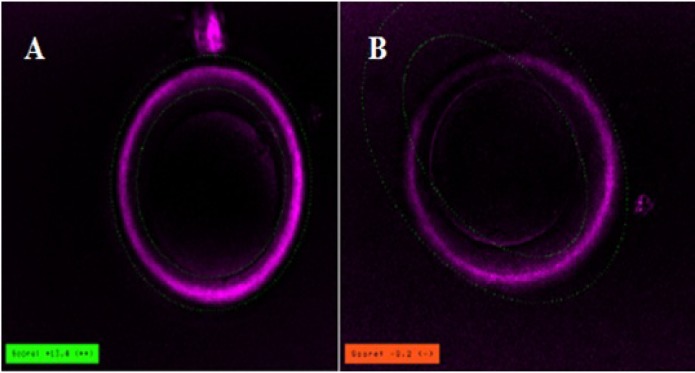
The inner layer of ZP of MΙΙ oocyte imaged with the Polscope. A) High ZP birefringent oocyte; B) Low ZP birefringent oocyte. ( ×40 magnification

With aid of Polscope, the MS could be visualized only in one of the IVM oocytes (Figure 2). However, the oocytes were divided into two groups based on the inner layer of ZP of MΙΙ oocyte: high/positively, and low/negative ZP birefringents. Finally, from these mature oocytes, 8 (61.5%) oocytes presented a high/positively scoring ZP and 5 (38.4%) with a low/negatively scoring ZP birefringents (Figure 3).

**Table I:** Oocyte yield and maturation rates in gynecologic patients who underwent IVM

**Pathology**	**Mean age (years)**	**Retrieved oocytes (n)**	**Mean retrieved oocytes**	**Discard oocytes (n)**	**Immature oocytes (n)**		**Matured oocytes (n)**		**IVM rate (%)**
					**GV**	**MI**	**24 hr**	**48 hr**	
Leiomyoma (n=7)	38.8±7.2	14	2±2.8	3	8	3	0	1	9.09
Squamous metaplasia (n=7)	38.3±6.6	16	2.5±2.07	7	6	3	1	1	22.2
Mucinous Cyatadenoma (n=4)	28.5±6.13	4	1±0.8	3	1	0	0	0	0
Endometric cystic (n=2)	34±15.56	1	0.5±0.7	0	0	1	0	0	0
Mature cystic teratoma (n=2)	33.5±4.94	5	2.5±3.53	3	1	1	0	0	0
Papillary serous cystadenocarcinoma (n=2)	28±2.82	4	2±1.4	0	3	1	0	0	0
Cervical neoplasma (n=2)	34±8.48	17	8.5±12.02	2	15	0	4	6	66.6

GV= germinal vesicle; MI= metaphase I; IVM= in vitro maturation

* mean±SD.

## Discussion

Under natural conditions, the ovaries of reproductive age patients contain a population of follicles in all growth stages. Oophorectomy can be performed for the purpose of fertility preservation in benign malignant diseases. Aspirating immature oocytes from ovaries is a milder treatment for infertility that caused by toxic chemotherapy. Furthermore, IVM technology does not require the use of large doses of gonadotropin for oocyte maturation. Also, it is feasible to reduce the risk of cancer recurrence rates. Moreover, the immature oocytes harvested from ovarian biopsies can be fertilized or vitrified after IVM. Therefore, IVM combined with the ovarian tissue freezing can be an additional strategy for fertility preservation (10). In this study, the immature oocytes collected from unstimulated ovaries showed a good developmental competence in gynecologic patients. Also, it has to be declared that almost half of our cases were over 38 years old. The IVM success rate that we achieved was 30.2%, which is higher than Wilken-Jensen *et al*. (2013) who reported only 3.1% maturation rate. In their study, 682 immature oocytes from surplus medulla tissue during cryopreservation of ovarian tissue were retrieved, but only 21 of them reached MII. They found that immature oocytes, obtained from ovary and subjected to a cooling period before recovery, had poor developmental competence (9). However, Imesch *et al*. (2013) showed a higher maturation rate of 61.9% (39/63) following IVM for up to 48 hr. Furthermore, the majority of these cases were non-cancerous patients (13). Fasano and associates (2011) and Escribá *et al*. (2012) also reported the maturation rates of 31% and 36.1%, respectively. They suggested that the major issue for decrease of IVM rate was delay in transport and oocyte collection from patients with oncological diseases (22-23). There are several factors for these different reports of IVM rates, such as source of oocytes (oncologic and nononcologic patients), IVM medium and technical procedures. Our data showed that the number of oocytes collected was negatively correlated with the patients’ age. This could be due to the experiment with less limitation for oophorectomy in aged patients compared to young women, similar to Fasano *et al*. (2011). They showed that for post-pubertal patients, there were no difference in the number of oocytes in patients who were less or over 30 year old (22). Conversely, Cha *et al*. (1998) reported that with advanced age, the number of immature oocytes that retrieved from an ovary were decreased (24). Also, Mohsenzadeh *et al*. (2012) showed that there was no significant relation between the rates of oocyte maturation and age of women. Their findings also demonstrated that IVM was useful for the majority of infertile cases (17).

Some studies have revealed that the quality of oocytes can be a determining factor in fertilization and embryonic developmental competence (19, 25). Oocyte is a complex cell with many organelles, such as MS and ZP that their dislocation or degeneration can decrease the oocyte viability (14). Omidi *et al*. (2013) and Nazari *et al*. (2011) demonstrated that following ovarian hyperstimulation of infertile women without any malignant disease, maturation rates were 65.9% and 59.4%, respectively. Also, most common anomaly in the IVM group was RF (15, 26). But in this study, wide PVS (36.3%) and dark cytoplasm (27.2%) were the most frequent abnormalities in IVM oocytes. In addition, fPB was noticed in some oocytes. Others observed that fPB was the most common anomaly in in vivo-matured oocytes (27, 28). Furthermore, Miao *et al*. (2004) concluded that fPB may be related to oocyte aging (29).

Analysis of oocyte morphological criteria, in combination with MS and the ZP visualization using the Polscope could be noninvasive and strong predictor of good quality oocytes. However, Braga *et al*. ( 2008) and Omidi *et al*. (2013) observed the visible MS in most of the IVM oocytes (15, 30). Conversely, in this study, MS could be visualized only in one of the IVM oocytes. Raju *et al*. suggested that the temperature, maternal age and in-vitro manipulations of oocytes can influence the MS kinetics and morphology (31). Visualization using the Polscope reveals the ZP as a three-layer structure of glycoproteins that forms during oogenesis. Moreover, Lasienė *et al*. (2009) demonstrated the thickness of the ZP had no influence on the embryo development after ICSI (32). Braga *et* *al*. (2010) and Petersen *et al*. (2011) reported the ZP birefringence in human oocytes cannot be negatively influenced by IVM (33, 34). Omidi *et al*. (2013) showed a high/positive and low/negative scores of ZP based on the birefringence of the inner ZP layer (15). In the present study, 8 (61.5%) of the oocytes presented a HB ZP, while 5 (38.4%) oocytes showed LB ZP. Also, the LB ZP was detected in abnormal IVM oocytes, similar to those reported by Omidi et al. (2013) (15).

## Conclusion

In conclusion, oocytes maturation post IVM from unstimulated ovaries seem to have good developmental potential in malignant and benign gynecologic patients. Therefore, further studies should be performed to advance the oocyte maturation program, such as co-culture system, for fertility preservation.

## Note

***This article extracted from M.Sc. thesis. (Fereshteh Safian)***
